# Magnetic particles with perpendicular anisotropy for mechanical cancer cell destruction

**DOI:** 10.1038/s41598-017-04154-1

**Published:** 2017-06-26

**Authors:** Rhodri Mansell, Tarun Vemulkar, Dorothée C. M. C. Petit, Yu Cheng, Jason Murphy, Maciej S. Lesniak, Russell P. Cowburn

**Affiliations:** 1 Cavendish Laboratory, University of Cambridge, JJ Thomson Avenue, Cambridge, CB3 OHE UK; 2 The Institute for Translational Nanomedicine, Shanghai East Hospital; The Institute for Biomedical Engineering & Nano Science, Tongji University School of Medicine, Shanghai, 200120 China; 3 Northwestern University Feinberg School of Medicine, 676 North Saint Clair Street, Suite 2210, Chicago, Illinois 60611 United States

## Abstract

We demonstrate the effectiveness of out-of-plane magnetized magnetic microdiscs for cancer treatment through mechanical cell disruption under an applied rotating magnetic field. The magnetic particles are synthetic antiferromagnets formed from a repeated motif of ultrathin CoFeB/Pt layers. *In*-*vitro* studies on glioma cells are used to compare the efficiency of the CoFeB/Pt microdiscs with Py vortex microdiscs. It is found that the CoFeB/Pt microdiscs are able to damage 62 ± 3% of cancer cells compared with 12 ± 2% after applying a 10 kOe rotating field for one minute. The torques applied by each type of particle are measured and are shown to match values predicted by a simple Stoner-Wohlfarth anisotropy model, giving maximum values of 20 fNm for the CoFeB/Pt and 75 fNm for the Py vortex particles. The symmetry of the anisotropy is argued to be more important than the magnitude of the torque in causing effective cell destruction in these experiments. This work shows how future magnetic particles can be successfully designed for applications requiring control of applied torques.

## Introduction

The ability of magnetic fields to safely penetrate the human body and interact with magnetic particles creates a promising route for novel therapies to destroy cancer tumours^1–4^. One proposed approach is to use magnetic particles that are mechanically actuated by an applied magnetic field^1, 2, 5–8^. Instead of the magnetic field causing the particle to heat up, as in the more common magnetic hyperthermia^9^, the field creates a torque on the magnetization which, through the magnetic anisotropy becomes a torque on the physical particle. This torque can be exploited in various ways – to trigger receptors associated with cell death^10^, open ion channels^5^ or deliver direct physical damage to cells^1, 11, 12^ – and has been shown to cause cell death both *in*-*vitro* ^1, 5^ and *in*-*vivo* ^3, 10^.

In order to deliver direct mechanical damage, the torque on the magnetization must translate to a force applied to the cell, which can damage the cell membrane^1, 6^ or even organelles if the magnetic particle is internalized by the cell^3, 11–14^. However, in order to improve the efficiency of magnetic particles in causing cell death, the applied torque should be optimized for the chosen magnitude and form (oscillating, rotating etc.) of the applied field. The key parameter here is the magnetic anisotropy of the particle which links the torque on the magnetization to a mechanical torque on the particle. An understanding of how torques can be efficiently created and controlled can also be applied to other situations where mechanical actuation is used such as cell manipulation, active materials or in microfluidics^15^.

In this paper the effectiveness of perpendicularly magnetized particles for causing cancer cell destruction is demonstrated. They are compared to Py vortex particles, a commonly used type of particle for mechanical cell destruction^1, 3, 5, 16^. Understanding the importance of the symmetry and magnitude of the magnetic anisotropy of the particle in applying a mechanical torque offers a route to highly efficient mechanical cell disruption.

## Magnetic particles

Two types of 2 μm diameter planar magnetic microdiscs were made by sputtering or thermal evaporation and lift-off (see methods) of magnetic thin films. Identical arrays each type of microdisc were fabricated on a Si wafer and magnetically characterized.

The first type of microdisc uses pairs of perpendicularly magnetized CoFeB layers coupled by Pt/Ru/Pt spacers to give an antiferromagnetic state at remanence (see methods for the full stack). This basic structure is then repeated twelve times in order to increase the total moment^17^, giving a total magnetic thickness of 21.6 nm.

**Figure 1:**
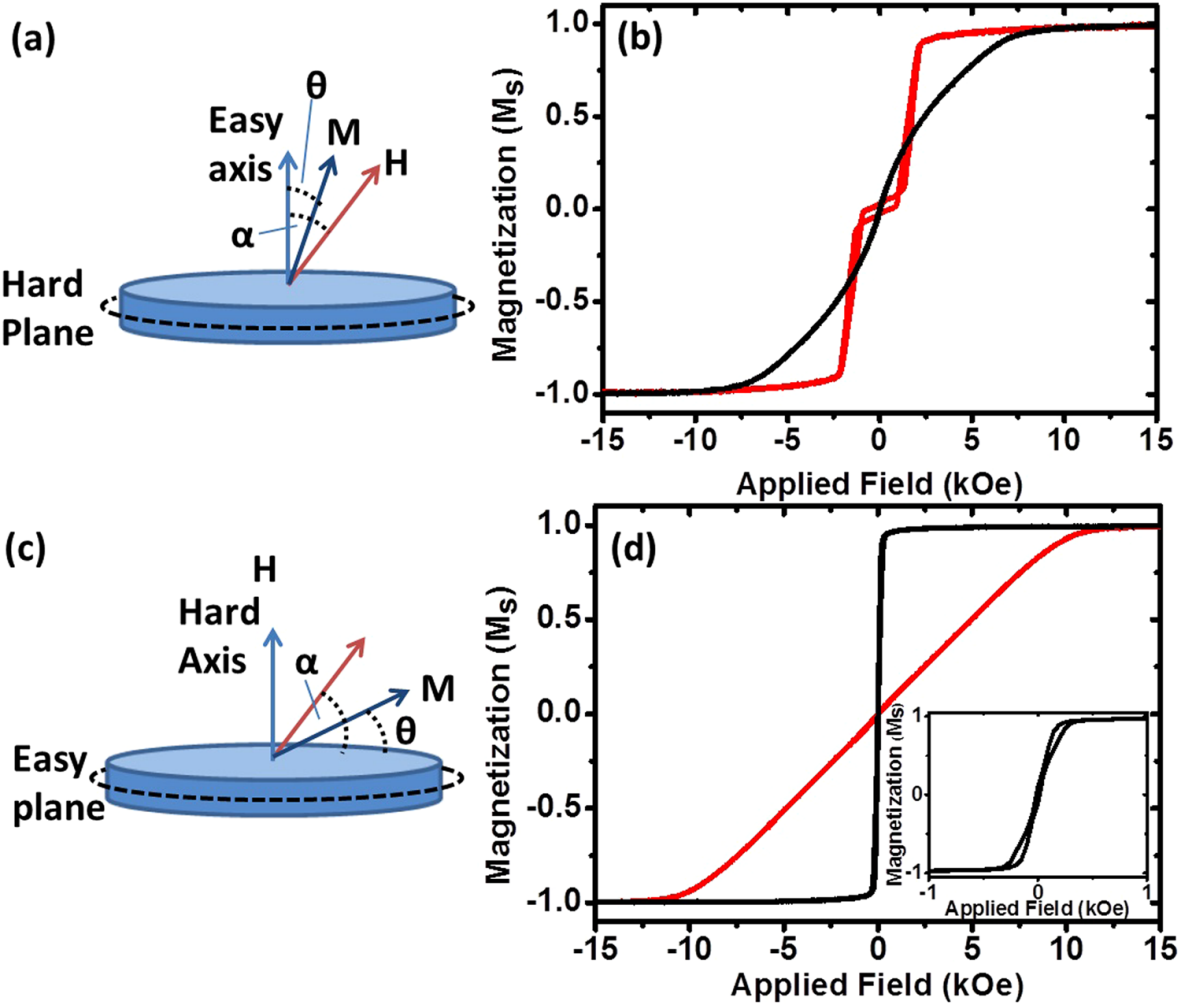
(**a**) Schematic of the angles describing perpendicularly magnetized particles and (**b**) out-of-plane (red) and in-plane (black) hysteresis loops for an array of 2 μm CoFeB/Pt particles. (**c**) Schematic of the angles describing in-plane magnetized particles and (**d**) out-of-plane (red) and in-plane (black) hysteresis loops for an array of 2 μm Py vortex particles. The inset in (**d**) is a zoom of the low field region showing the vortex magnetization behavior.

Figure 1(a) shows a schematic of a disc with an out-of-plane easy direction. Figure 1(b) shows vibrating sample magnetometry (VSM) data from the array of 2 μm CoFeB/Pt microdiscs patterned on a wafer. The red curve shows the response to a field applied along the out-of-plane easy axis. Below a field of around 2 kOe the antiferromagnetic coupling causes one of each pair of layers to align antiparallel to the other leading to little net magnetization in the disc. The black curve is for a field applied in the plane of the disc, along the hard axis, which shows saturation at around 9 kOe. The hard axis saturation field for the CoFeB/Pt microdiscs is a measure of the applied field necessary to overcome both the perpendicular magnetic anisotropy energy and the RKKY coupling energy. It is given by *H*_*Sat*_ = *H*_*K*_ + 2*H*_*J*_, where *H*_*K*_ is the anisotropy field, and *H*_*J*_ is the effective coupling field. The coupling field can be derived from the easy axis loop and is around 2 kOe for the sample shown in Fig. 1(c) ^18^. This yields an anisotropy field *H*_*K*_ of 5 kOe.

The second type of particle is the Py vortex disc^1, 19^. The demagnetization energy of the 60 nm thick Py layer with weak intrinsic in-plane anisotropy causes the magnetization to curl round following the edge of the particle. The in-plane magnetic response for an array of 2 μm Py microdiscs shown by the black curve in Fig. 1(d) has zero net moment at zero field (see the inset in Fig. 1(d)) due to the formation of the vortex state. The out-of-plane hard axis (in red) shows saturation at an anisotropy field, *H*_*K*_, of 10 kOe, which in the case of the Py discs is a direct measure of the anisotropy energy that prefers to orient the magnetization in the plane of the microdisc.

These two sets of magnetic particles have some similar properties, they have no magnetization at remanence, and similar hard axis saturation fields. Crucially however, they differ in the symmetry of the magnetic anisotropy. The CoFeB/Pt microdiscs have an easy out-of-plane magnetization axis, while the Py microdiscs have an easy magnetization plane due to the weak in-plane anisotropy of Py.

## ***In*-*vitro*** experiments

**Figure 2:**
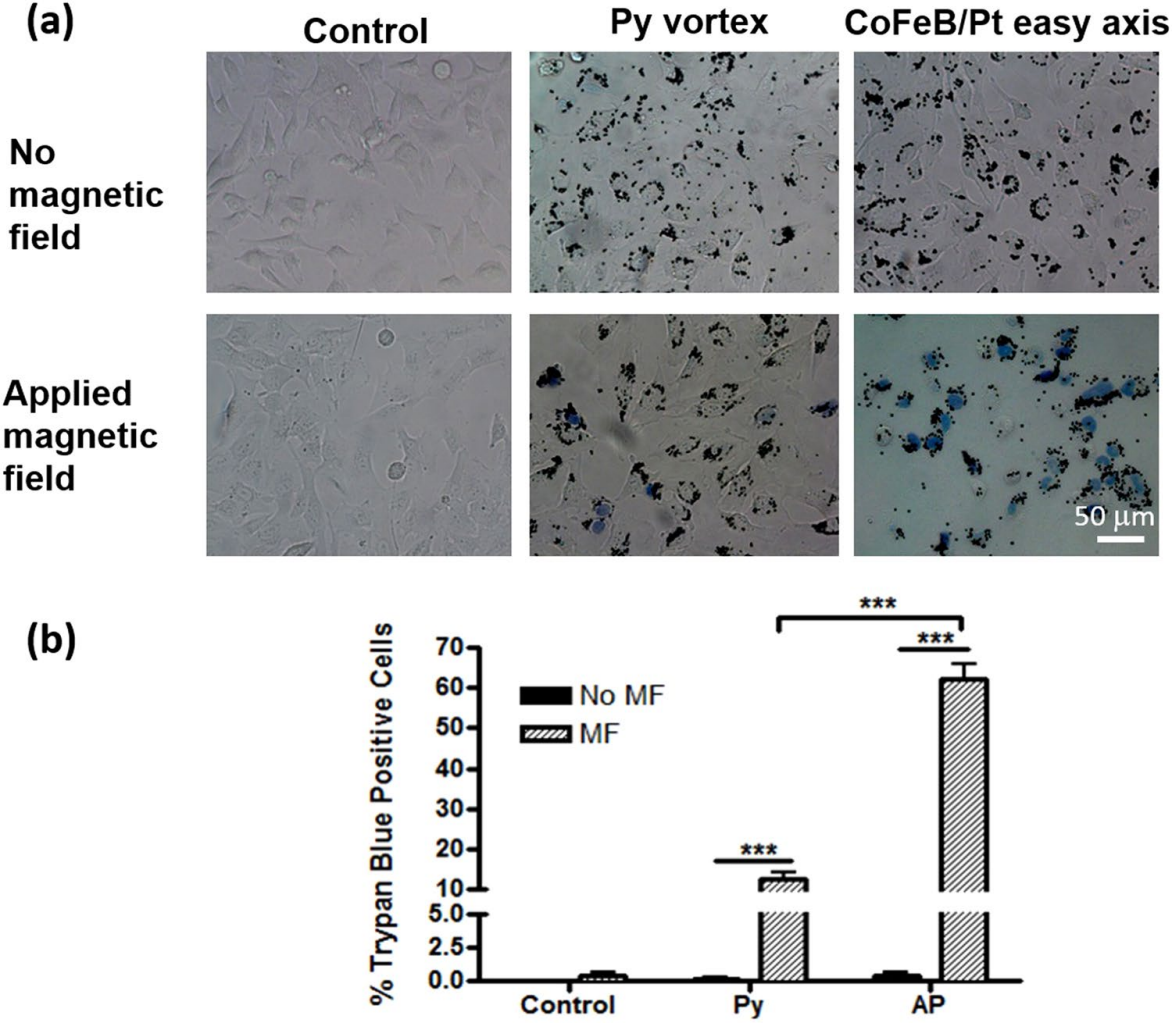
(**a**) Differential interference contrast images of U87 cells after trypan blue staining. U87 cells were incubated with growth media (control), Py particles and AP particles for 24 hours respectively. Cells were then incubated with trypan blue solution (0.2% in PBS) and received magnetic field treatment (MF) for 1 minute. (**b**) Quantification of trypan blue positive cells after 1 minute MF. Data are presented as mean ± standard error (n = 5 images). ***p < 0.001 (ANOVA).

To test the effectiveness of these two types of particles for cancer cell destruction they were incubated with human U87 brain tumor cells for 24 hours at a concentration of 50 particles per cell (see methods for details). Whilst both sets of particles have gold caps on either side, these were not functionalized for specificity. However, the particles are internalized by the cells (see supplementary information for TEM images)^3, 13^ during the incubation period. The incubated cells and particles are then subjected to a 10 kOe rotating field for one minute. After the applied field treatment a trypan blue staining is carried out which stains cells which have compromised cell membranes. The cells are then imaged by optical microscopy. Representative images of this experiment are shown in Fig. 2(a) and summary statistics based on counting cells in five images are given in 2(b).

The control groups of cells account for both the field treatment, as well as the particle incubation. Cells with no particles that were either given or withheld the applied field treatment, and cells incubated with each particle type but were withheld the magnetic field treatment, all showed negligible amounts of staining.

With a magnetic field applied, the cells incubated with the Py vortex particles show 12 ± 2% staining, whilst those incubated with the CoFeB/Pt particles show 62 ± 3% cell death or damage. It is therefore important to understand the cause of this divergence in effectiveness. Two main distinctions between the particles can be considered - the difference in the symmetry of the anisotropy and the difference in magnitude of the torque.

## Symmetry considerations

**Figure 3:**
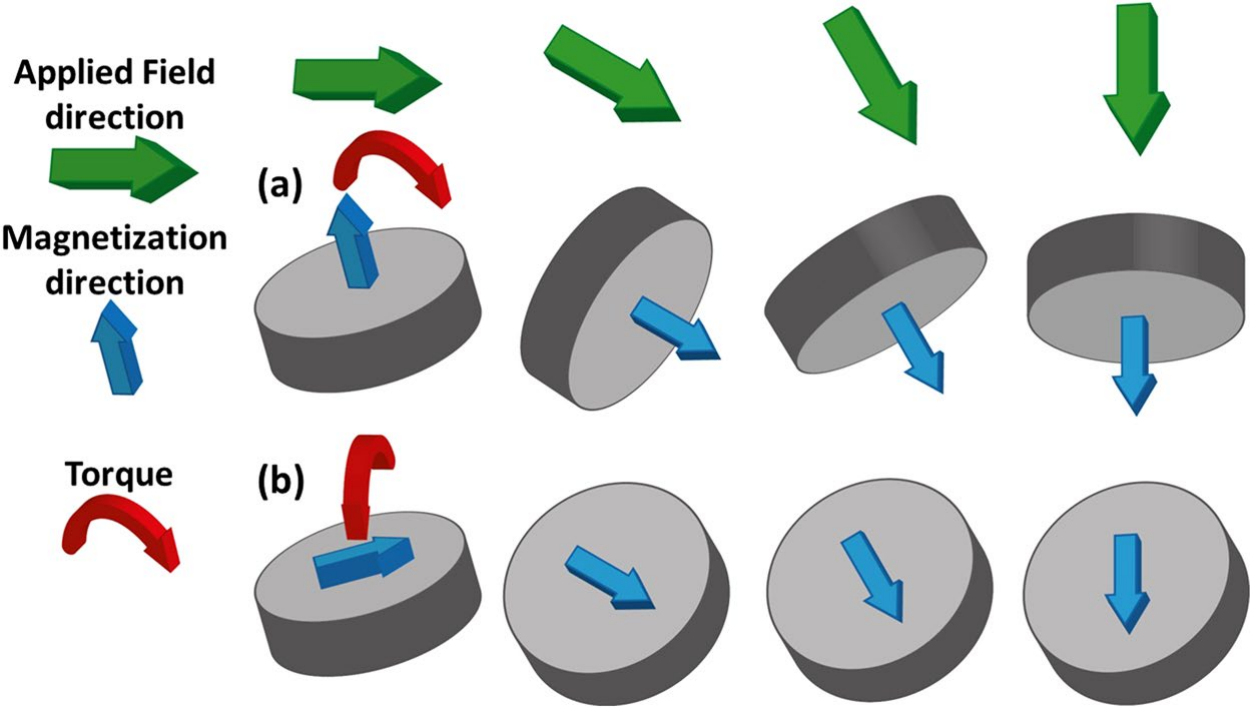
Schematic showing the magnetization direction and torques on (**a**) perpendicular particles (upper row) and (**b**) Py vortex particles (lower row) under an applied rotating field.

In Fig. 3 the difference in behavior of the two types of particle is shown schematically. For the CoFeB/Pt particles (Fig. 3(a)), the 10 kOe applied field causes the easy axis of the particle to align with the field and then the easy axis will rotate with the field because of the uniaxial out of plane anisotropy. The behavior of the Py vortex particles is rather different (Fig. 3(b)). Any initial angle between the easy plane of the particle and the magnetic field will lead to an out-of-plane canting of the magnetization and associated torque on the particle, whose effect is to align the easy plane to the field direction. Since a rotating field is used in the experiments discussed here, such an angle between the easy plane and the magnetization will exist until the easy plane is aligned to the plane of the rotating field. However, once this has occurred the in-plane magnetization can rotate with the applied field without exerting a further torque on the particle. Therefore, the underlying symmetry of the anisotropy will cause a substantial difference in effectiveness between the two particles.

## Estimating magnetic torques for the two particles

The other possible reason for the difference in killing effectiveness between the types of particle is the magnitude of the torques applied on the particles. It is helpful to first approximate the torque applied by the particles using a simple Stoner-Wohlfarth like model for the magnetization under an applied field. The validity of using the model is then confirmed via experimental data.

The magnetic torque is given by ***T*** = ***m*** × ***H***, where m is the magnetic moment and H the applied field. However, a torque on the magnetization does not necessarily translate to a torque on the physical particle. It is the magnetic anisotropy which links the magnetization and physical particle. We model this interaction using a quasi-static Stoner-Wohlfarth-like model^20, 21^. Usually, dynamic models are used^4, 14^, but the large torques involved here mean that the retarding effect of the fluid drag is negligible (see supplementary material) and so the particle is assumed to be able to reach the minimum energy configuration on the timescales of the experiment. In this model the energy for a magnetic disc of moment m is given by:

1 $$E=-Hm\,\cos (\alpha -\theta )+\frac{{H}_{k}m}{2}{\sin }^{2}\theta ,$$

where H is the applied field, H_k_ is the anisotropy field, α is the angle of the applied field relative to the easy axis and θ is the angle of the magnetization relative to the easy axis. See Fig. 1(a) and (c) for the definitions of α and θ for the two sets of particles. From this energy equation and the definition of torque the model gives a simple formula for the maximum torque (see supplementary material): ${T}_{max}=\frac{{H}_{k}m}{2}$, which can be achieved as long as the applied field is greater than $\frac{{H}_{k}}{\sqrt{2}}$. This maximum torque is easily estimated for both particles.

For a Py vortex microdisc the magnetic moment is 1.5 × 10^−10^ emu (1.5 × 10^−13^ Am^2^) and the anisotropy field 10.8 kOe giving a maximum torque of 7.5 × 10^−7^ dyn.cm (7.5 × 10^−14^ Nm). For a CoFeB/Pt particle the moment is 0.75 × 10^−10^ emu (7.5 × 10^−14^ Am^2^), half that of the Py vortex particles due to the reduced thickness of the magnetic material in these particles. The anisotropy field H_k_ of the CoFeB/Pt microdiscs is 5 kOe, again half the value found for the Py particles. The maximum torque for the CoFeB/Pt particles is then 1.8 × 10^−7^ Dyn.cm (1.8 × 10^−14^ Nm). These torques are equivalent to forces of 75 nN for the Py vortex particles, and 18 nN for the CoFeB/Pt particles.

## Experimentally measuring torques

The estimated torques that we derived from the Stoner-Wohlfarth equation may be validated experimentally by measuring the magnetization angle as a function of field angle. We do this for a series of applied field strengths, for both CoFeB/Pt and Py. Anomalous Hall effect (AHE) measurements for the CoFeB/Pt and anisotropic magnetoresistance (AMR) measurements for the Py are performed on Hall bars^22^ patterned from thin films that are nominally identical to those used to fabricate the CoFeB/Pt and Py microdiscs characterized in Fig. 1. The torque as a function of field angle is also simulated using a Stoner-Wohlfarth like model and compared to the experimental data (see Supplementary Information).

### CoFeB/Pt microdiscs

**Figure 4:**
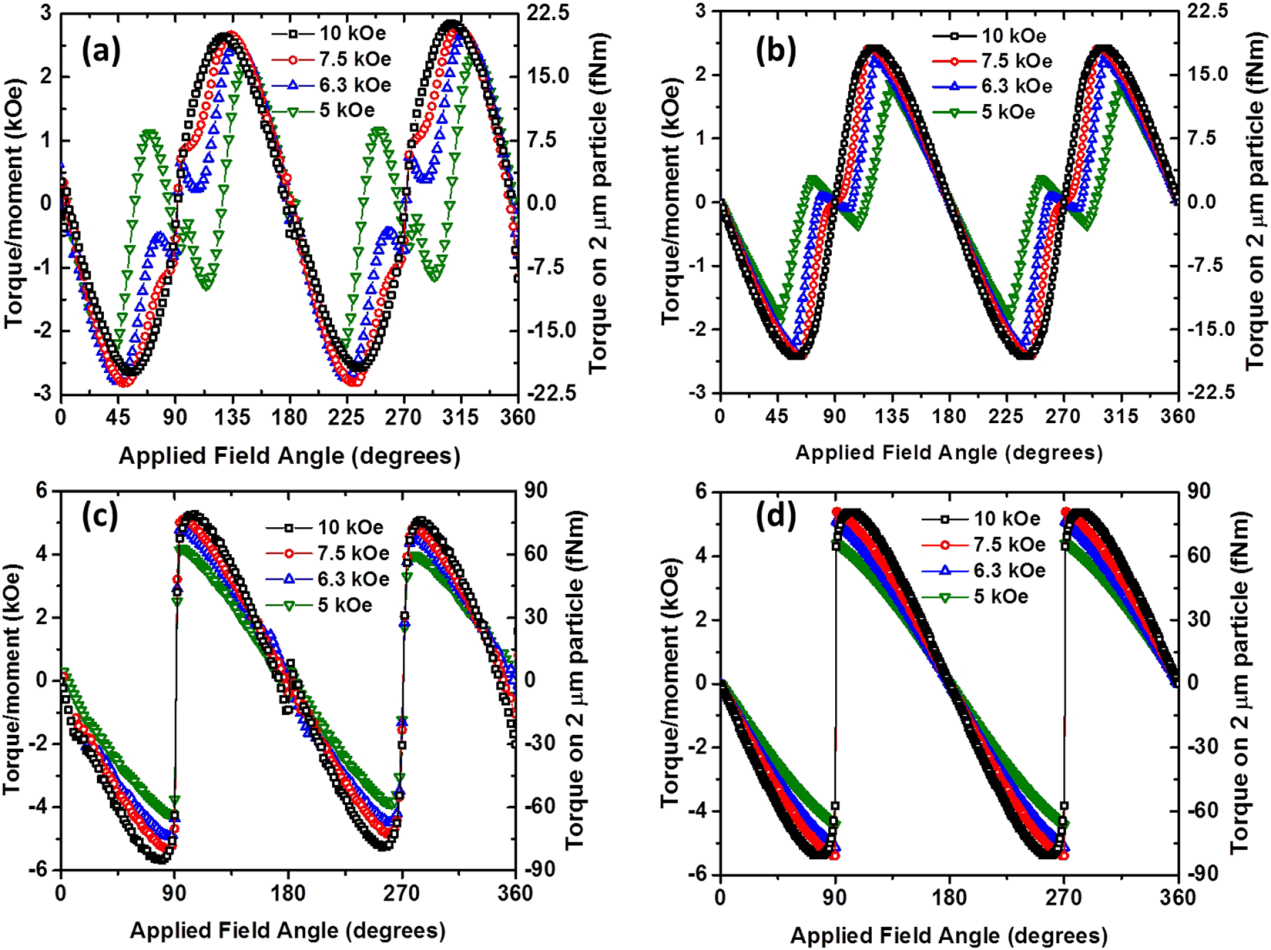
(**a**) Calculated torque per unit moment from anomalous Hall effect measurements as a function of applied field and angle for a CoFeB/Pt thin film. The right hand axis gives the torque assuming the moment of a 2 micron diameter particle. (**b**) Simulated torque per unit moment using the magnetic characteristics of the sample measured (**a**). (**c**) Calculated torque per unit moment from anomalous magnetoresistance measurements as a function of applied field and angle for a Py thin film. (**d**) Simulated torque per unit moment using the magnetic characteristics of the sample measured in (**c**).

For the CoFeB/Pt film the normalized AHE data is used to extract the magnetization angle as a function of applied magnetic field angle, carried out for a series of different rotating field strengths. This can then be used to calculate the torque per unit moment since *T*/*m* = *B* sin(*α* − *θ*). This extracted torque is shown in Fig. 4(a), with simulated torque per unit moment shown in Fig. 4(b), with the equivalent torque for a two micron size disc shown on the right-hand scale. The simulation uses two RKKY coupled layers with a Stoner-Wohlfarth like anisotropy (see supplementary information for details of the model). The simulation assumes a uniform distribution of RKKY coupling values from 1.8 erg/cm^2^ to 3.4 erg/cm^2^ in order to match the easy axis hysteresis loop of a thin film that is nominally identical to those used to fabricate the microdiscs for the cell experiments and measured in Fig. 1(b). Notably, the torque extracted experimentally for the 10 kOe applied field matches closely the simulated data. The Stoner-Wohlfarth approach predicts the maximum torque exerted by the CoFeB/Pt microdiscs which is experimentally found to be around 20 pNm, compared to the 18 pNm predicted. Further, features such as the reversal in the sign of the torque around the hard axis (applied field angle of 90°) for the lower applied fields are found in both the experimental and theoretical plots. The magnitudes of the torques around the hard axis differ between experiment and simulation, which is due to the RKKY coupling causing the moments to scissor which cannot be distinguished experimentally from rotation of the moment (see supplementary material). Also, jumps in the magnetization are seen experimentally around the hard axis, which are not seen in the minimum energy simulations. This is to be expected as the perpendicular materials tend to switch across the hard axis by domain processes that are not modelled by the minimum energy approach.

### Py vortex microdiscs

Figure 4(c) and (d) are the experimentally measured and simulated torque for the Py thin film (see supplementary material for more detail). The simulated and experimental data closely match each other. The sharp change in the torque at the applied field angle of 90° corresponds to the magnetization jumping since the applied field is not high enough to overcome the 10.8 kOe anisotropy field necessary to saturate the discs in the hard axis direction. Most importantly, the magnitude of the torque measured is that predicted by the Stoner-Wohlfarth model which proves to be a robust method for predicting the behavior of the Py microdiscs in an applied field. This value agrees closely with that estimated from the Stoner-Wohlfarth model at around 75 pNm.

## Discussion

From the measurement and simulations of the torque applied it is clear that the Py vortex discs can apply a much larger maximum torque than the CoFeB/Pt microdiscs. Since the CoFeB/Pt microdiscs are much more effective at killing cells, this shows that in this application, it is the symmetry considerations which are most crucial. With a rotating field, a microdisc with a uniaxial anisotropy ensures that a torque is continuously applied rather than a transitory effect which only occurs at the introduction of the field as is the case for a microdisc with an easy magnetization plane.

There are three other mechanisms to consider that may cause the cell death seen here. Firstly, there is magnetic hyperthermia, however, the relatively slow rotation frequency means that heating will be fairly negligible here (see supplementary information). Secondly, the initial magnetization of the particles will create dipole moments which cause the particles to agglomerate. By modelling the stray fields from the particles, the field gradient, and so the force on the particles, can be calculated. For both sets of particles the maximum force is in the nanoNewton range, comparable to the torques on the particles (see supplementary material). However, due to the difference in effective cell death we can rule out this mechanism, at least for the CoFeB/Pt particles. Thirdly, applying the magnetic field for a longer time has been shown to lead to more effective killing for the Py particles^1, 3^. Particle agglomeration, for instance, may lead to an effective net anisotropy that causes the particles to rotate under the field. It is clear that the simple physical analysis applied here will only be able to capture part of the interaction of the particles with a biological system.

These results show the particular importance of controllably engineering the magnitude and symmetry of the magnetic anisotropy when applying torques on soft matter systems using magnetic particles. The scale of the torque can be modified by changing either the moment, or through controlling the anisotropy, both of which can be achieved in the CoFeB/Pt system due to its repeat structure and interface-controlled anisotropy (see also supplementary material). This provides a method for reliably controlling the maximum torque that is exerted by particles under a rotating field. For particles with large moments and anisotropies under large applied fields the type of quasi-static model used here is most relevant. It may be that for cancer cell destruction applications the large torques used here are not beneficial, if they tend to promote necrosis over apoptosis. However, finding the most appropriate torque also requires knowledge of the exact mechanisms of damage, which is beyond the scope of this paper. The relevance of these results extends beyond the materials system studied here. One major conclusion is that in a rotating field the most efficient torques can be exerted by particles with a uniaxial anisotropy. Furthermore, it is possible to design through shape anisotropy, and dipole or RKKY coupling, particles with zero remanent moment and anisotropies of the order of those used here^23^. This allows other materials to be considered, such as magnetite, which may be preferred for *in vivo* applications. The control over the torque which can be achieved with precisely engineered magnetic particles will allow them to be used not only for therapeutic applications, but also for microrheology studies, materials actuation in areas such as soft robotics, and the manipulation of cells and cellular components. Applications where the initial orientation of the particles can be controlled, in particular, will be able to exploit the large torques demonstrated here.

## Conclusion

The effectiveness of perpendicularly magnetized synthetic antiferromagnets is demonstrated for *in vitro* cell killing for cancer therapeutic applications. 2 μm particles made from such multilayers were able to kill 62% of cells when placed in a 1 T rotating field for one minute, where Py vortex particles could only kill 12% of cells under identical conditions. By using large applied fields the anisotropy of the particles can be exploited to produce large forces in the nanoNewton range. By understanding the importance of the symmetry and strength of the anisotropy, magnetic particles can be better designed, and new materials exploited for the desired application.

## Methods

The CoFeB/Pt multilayers were made by magnetron sputtering and consisted of Au (5 nm)/[Ta (2 nm)/Pt (2 nm)/CoFeB (0.9 nm)/Pt (0.3 nm)/Ru (0.9 nm)/CoFeB (0.9 nm)/Pt (2 nm)]_12_/Au (5 nm). The Py vortex particles were thermally evaporated with a layer structure consisting of Au (5 nm)/Py (60 nm)/Au (5 nm). For the vibrating sample magnetometry data in Fig. 1, thin films of the two materials were patterned in to 2 micron pillars using ma-N 1410 resist as a milling mask.

For the particles used in Fig. 2, the growth was carried out onto 2 micron wide pillars of ma-N 1410 and the material grown on the pillars was lifted off into solution. The particles were washed in deionized water. The U87 human glioma cell line was purchased from the American Type Culture Collection (Manassas, VA., USA). It was cultured in Dulbecco’s Modification of Eagle’s Medium (DMEM) (Mediatech Inc., Manassas, VA, USA), containing 2% penicillin and streptomycin antibiotic (Cellgro, Mediatech, Inc., Manassas, VA, USA) and 10% fetal bovine serum (FBS; Atlanta Biologicals, Lawrenceville, GA, USA). Magnetic particles were incubated with cells at a ratio of 50 particles per cell for 24 hours before magnetic field treatment. The cells were then incubated with trypan blue (0.2% in phosphate-buffered saline), placed in the centre of the field and the magnet was rotated for one minute. The 1 T field was provided by a circular Halbach array magnet (Bunting Magnetics Europe Ltd., Hertfordshire, UK) which is connected to a motor and rotated at 20 Hz. After the magnetic field treatment the cells were washed three times in phosphate-buffered saline then imaged using optical microscopy. The cell images were taken at 20x magnification. The blue cells and unstained cells were then counted for quantification. Five replicates were used per condition and a two-way ANOVA analysis was used to determine the p-values.

For the AHE and AMR measurements, thin films were patterned into 80 μm wide Hall bars using ma-N 1410 resist as a milling mask. The measurements were then conducted using a lock-in measurement with an ac current of 500 μA.

### Data Availability

The underlying data for this study are available at the University of Cambridge data repository, at https://doi.org/10.17863/CAM.9594, or from the corresponding author on reasonable request.

## Electronic supplementary material

Supplementary information
