# Supplementary material for: Magnetic particles with perpendicular anisotropy for mechanical cancer cell destruction
Source: Sci Rep. 2017 Jun 26;7:4257. doi: 10.1038/s41598-017-04154-1 (PMC5484683; doi:10.1038/s41598-017-04154-1)
Supplement: Supplementary file 1 — Supplementary information [file 41598_2017_4154_MOESM1_ESM.pdf]

Magnetic particles with perpendicular anisotropy for mechanical cancer cell destruction

Supplementary information

Rhodri Mansell[1]*, Tarun Vemulkar[1], Dorothée C.M.C. Petit[1], Yu Cheng[2], Jason Murphy[3], Maciej S. Lesniak[3], Russell P. Cowburn[1].

[1] Cavendish Laboratory, University of Cambridge, JJ Thomson Avenue, Cambridge, UK, CB3 OHE

[2] The Institute for Translational Nanomedicine, Shanghai East Hospital; The Institute for Biomedical Engineering & Nano Science, Tongji University School of Medicine, Shanghai, 200120, China

[3] Northwestern University Feinberg School of Medicine, 676 North Saint Clair Street, Suite 2210, Chicago, Illinois 60611, United States

*Correspondence to rhodri.mansell@aalto.fi

1. Particle internalization and effects of dose

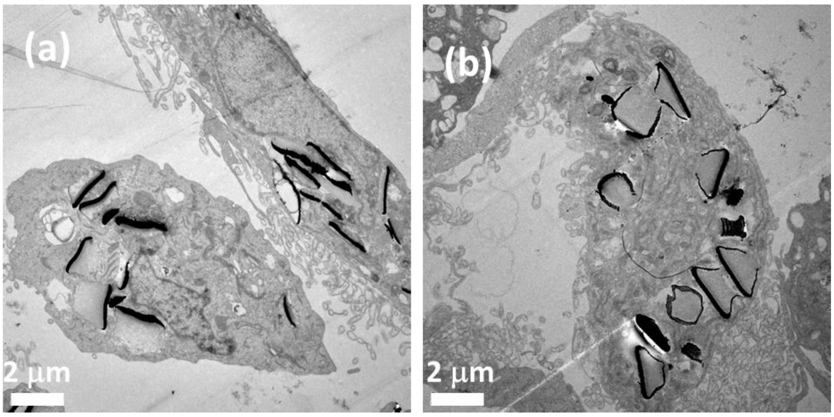

Figure S1. TEM imaging of (a) Py vortex particles and (b) CoFeB/Pt particles internalized into cells.

Figure S1 shows both types of particles after having been internalized into cells after 24 hours incubation starting with 20 particles per cell. In order to take the images cells are dehydrated and encased in a spur resin. They are then cut into roughly 90 nm slices for imaging. Previously, for identical Py vortex particles, the uptake in U87 glioma cells was estimated at 40±15 particles per cell when incubated with 50 particles per cell (as used in the experiments in the main paper) [S1], and due to the similar size and Au coating a similar uptake is expected for the CoFeB/Pt particles.

In figure S2 we show the survival of cells with different initial concentrations of particles per cell for the CoFeB/Pt particles. Cultures with different particle concentrations of 10 per cell, 20 per cell and 50 per cell, were incubated for 24 hours. The samples that underwent a magnetic field treatment were then subjected to a 1 T field rotating a 20 Hz for 30 minutes. The cell survival is estimated 24 hours after the application of the field using an MTT assay, with three replicates per condition. It is clearly

seen that with increasing numbers of particles per cell there is a lower survival both with and without a magnetic field. Due to the different assay and timing used here compared to the main text the survival rates differ for the no magnetic field case. In addition, the magnetic field is applied for a much longer time here (30 minutes) compared to the main text (1 minute) which may also contribute to the reduced survival for the magnetic field case. The data for 50 particles per cell show slightly lower survival without magnetic field to previously published data on Py vortex particles using the same protocol (CoFeB/Pt 68 %, Py vortex 78 %), whilst showing significantly lower survival in the case of an applied magnetic field (CoFeB 7 %, Py 38 %) [S1].

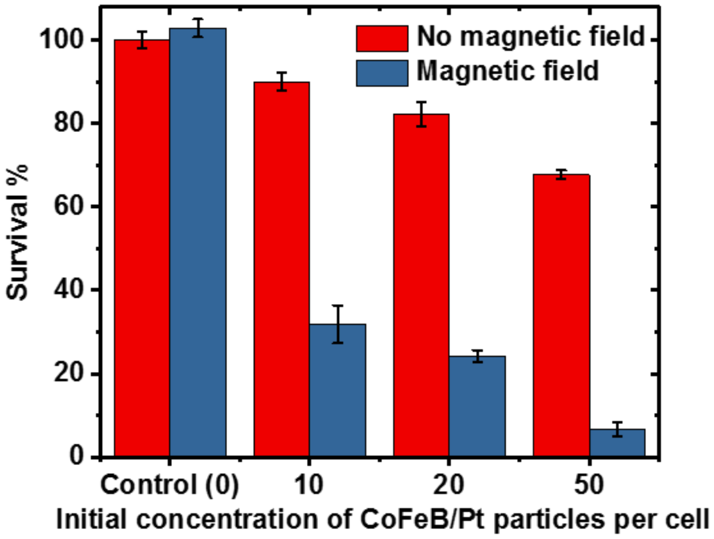

Figure S2. MTT assay of particle for various initial concentrations of CoFeB/Pt particles per cell, without magnetic field and with a 1T rotating magnetic field applied for 30 minutes.

2. Simulated and experimentally measured torques

The experimentally calculated torques are derived from measurements of the magnetization as a function of applied field angle on thin films. The films are nominally identical to those used to fabricate the microdiscs used in the cell experiments, and are patterned into Hall bars for these measurements.

Py vortex microdiscs

In order to model the behaviour of the Py vortex microdiscs we use a simple Stoner-Wohlfarth model which assumes there is only an anisotropy term along the direction between the hard and easy axes or planes. This is shown schematically in figure 1 (c) in the main manuscript. There is no anisotropy associated with the azimuthal angle, which is therefore neglected.

The energy density of the in-plane Py magnetic disc is therefore written as:

$$\frac{E}{V} = -HM_s \cos(\alpha - \theta) + K\sin^2\theta,$$

where H is the applied field, K is the magnetic anisotropy given by $K = \frac{H_k M_s}{2}$, M_S is the saturation magnetization, α is the angle of the applied field relative to the easy axis and θ is the angle of the applied field relative to the easy axis, as shown in figure 1 (c) in the main manuscript. Taking $\frac{dE}{d\theta} = 0$, the energy minimum is given by:

$$HM_s \sin(\alpha - \theta) = H_k M_s \sin\theta \cos\theta$$

Or

$$\sin(\alpha - \theta) = \frac{H_k}{H} \sin\theta \cos\theta$$

Now using the definition of magnetic torque $\boldsymbol{T} = \boldsymbol{m} \times \boldsymbol{H}$, the magnitude of the torque can be written as $T = mH \sin(\alpha - \theta)$, which can then be substituted in above to give

$$T = mH_k \sin\theta \cos\theta$$

Therefore in this model the torque can simply be described by the magnetization angle. This emphasises the role of the magnetic anisotropy in the creation of a torque. Further, it is easy to see that the maximum torque occurs when θ = 45°, which leads to a maximum torque of $T_{max} = \frac{H_k m}{2}$. However, in order for the torque to be maximized, the minimum field strength required is $\frac{H_k}{\sqrt{2}}$, which leads to the magnetization being at 45° when the field is aligned along the hard axis. We use the above equation to estimate the maximum torque exerted by the CoFeB/Pt microdiscs as well.

For the Py system, the parameters used in the simulations are the nominal value for the saturation magnetization of Py of M_S = 860 emu/cm^3, K = 4.65 x 10^6 erg/cm^3, and the film thickness is 60 nm. Figure S3 (a) and (b) shows the simulated and measured anisotropic magnetoresistance (AMR) data. For the simulation, the value of the AMR is assumed to be proportional to $\cos^2\theta$ [S2].

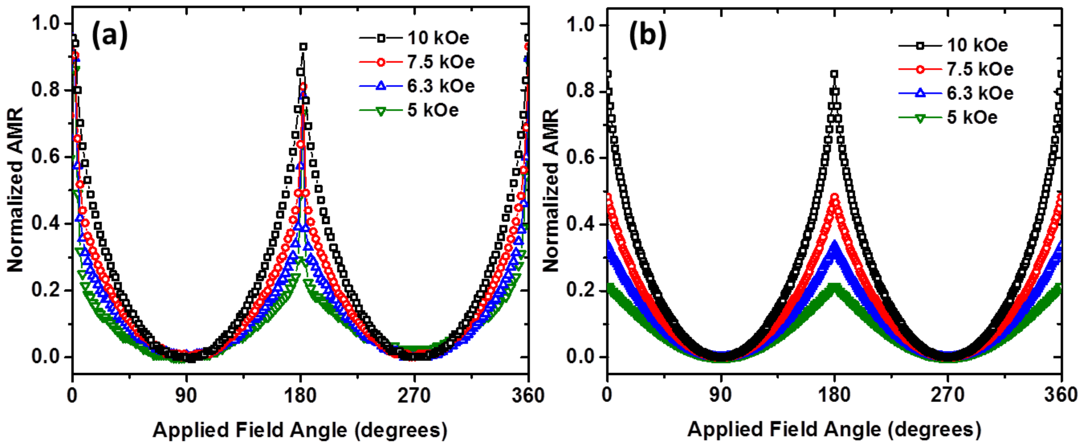

S3. The (a) experimental and (b) simulated AMR data for the Py system.

CoFeB/Pt microdiscs

For the CoFeB/Pt disc the equations have to be modified to include the RKKY coupling term. We assume the system can be modelled as two coupled layers giving:

$$\frac{E}{V} = -HM_s(\cos(\alpha - \theta_1) + \cos(\alpha - \theta_2)) + K(\sin^2\theta_1 + \sin^2\theta_2) + \frac{J}{t}\cos(\theta_1 - \theta_2)$$

Where θ_1 and θ_2 are the magnetization angles of the two layers and J is the RKKY coupling strength in energy per unit area and t is the thickness of the layer. This equation was then solved by finding the values of θ_1 and θ_2 that minimize the energy.

3. Modelling the CoFeB/Pt particles

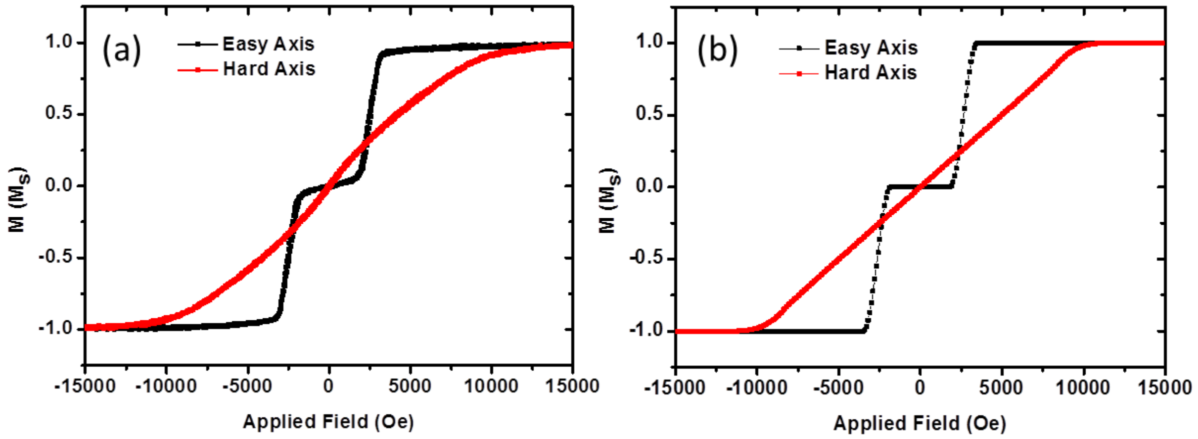

Figure S4. (a) Easy and Hard axis VSM loops of a 12 times repeated RKKY coupled CoFeB/Pt stack nominally identical to the one described in the main text. (b) The easy and hard axis loops given by the Stoner-Wohlfarth model used here.

In order to model the behaviour of the CoFeB/Pt particles we use the Stoner-Wohlfarth approach outlined above. By finding the magnetization angles which minimize the Stoner-Wohlfarth equation for a given field strength and direction, the torque can be found. From the hysteresis data shown in figure S4(a) various parameters for the model are extracted.

Ms	1100 emu/cm^3
Hard axis saturation field	9880 Oe
Coupling field	2540 Oe
Coupling energy (J)	0.25 erg/cm^2
Anisotropy field (H_k)	4840 Oe
Anisotropy energy (K)	2.66 x 10^6 erg/cm^3
Thickness, t	0.9 nm

In order to more accurately match the experimental data the coupling field was assumed to consist of a uniform spread of values from 0.18 erg/cm^2 to 0.34 erg/cm^2, centred around the experimentally determined value of 0.25 erg/cm^2.

4. Determining torques in the CoFeB/Pt system

Experimentally the torque is extracted by taking the normalized AHE signal and converting it into an angle by assuming that $\frac{M_z}{M_{tot}} = \cos\theta$, where M_z is the moment in the out-of-plane direction, M_{tot} is the total magnetization and theta is the magnetization angle. However, due to the RKKY coupling the magnetic layers lie in a scissored configuration around the hard axis of the disc. This range over which significant scissoring occurs, and the degree to which the layers diverge from each other depends on the magnitude of the applied field. Figure S5(a) shows the difference in angle between the two RKKY coupled layers in the simulation. It can be seen that for a range of fields around the hard axes at 90 and 270 degrees the two layers indeed diverge, quite substantially for the 5 kOe rotating field. This leads to a significant reduction in the value of M_{tot} for this range of field angles. Experimentally, we are unable to distinguish changes in M_{tot} from changes in angle, however. We can model this effect by treating the simulated data in a similar way to the experimental data. Instead of directly calculating the torque we can simulate the AHE data and then extract the torque. This extracted torque is shown in S5(b) and it can be seen that this very closely matches the experimental data shown in the main paper. A couple of important points can be made. Firstly, the peak magnitudes of the torques do not change. This is because the divergence in angle between the two layers is small at the applied field angles where the torque is greatest. Secondly, the greatest effect of the divergence of the two layers on the extraction of the torque is to exaggerate the torque for the lower applied field amplitudes around the hard axis, best seen in the 5 kOe data.

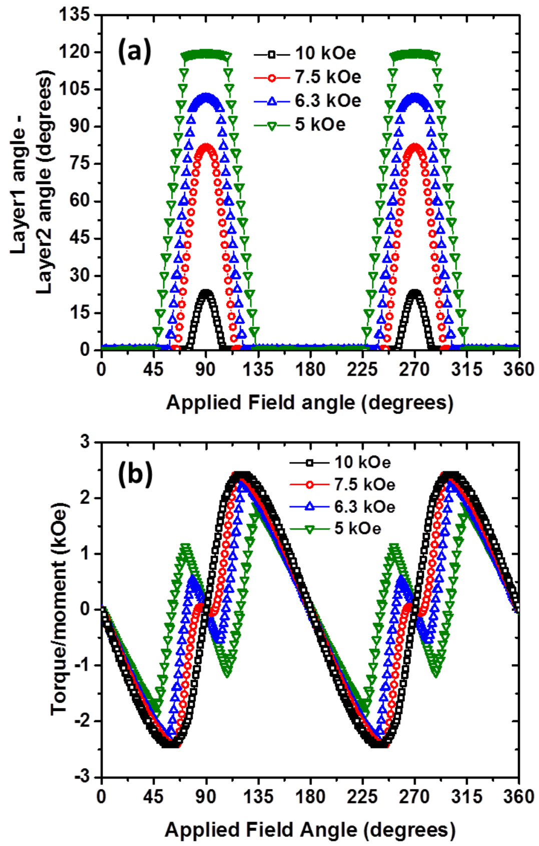

Figure S5. (a) Simulated divergence between the two RKKY coupled layers. (b) Simulated torque per moment which copies the experimental data extraction.

5. The effect of the anisotropy and the coupling field on the torque in the CoFeB/Pt system

The torque exerted by the CoFeB/Pt microdiscs is dependent on the magnitude of both the anisotropy, and the RKKY coupling in the discs. From figure S6(a) it can be seen that the maximum reduced torque for a PMA SAF microdisc is achieved as long as $H > \frac{H_K}{\sqrt{2}}$.

From figure S6(b) we see that the coupling field can also in fact have an important effect on achieving the maximum torque for the system. If the coupling field is too high, then, even at angles close to the easy axis, the two moments diverge away from each other to minimize the energy lost to the antiferromagnetic interlayer exchange coupling. This prevents the system from reaching the maximum torque condition. Thus, while the RKKY coupling does not cause a torque to be exerted on the particle because it is not linked with the physical frame of the particle, it can still cause a significant reduction in the maximum torque achievable by affecting the orientation of the angles in the applied field. We see that for our system, as long as the coupling field is below roughly a third of the applied field, then the maximum torque can still be achieved and this is an important consideration during the thin film fabrication process.

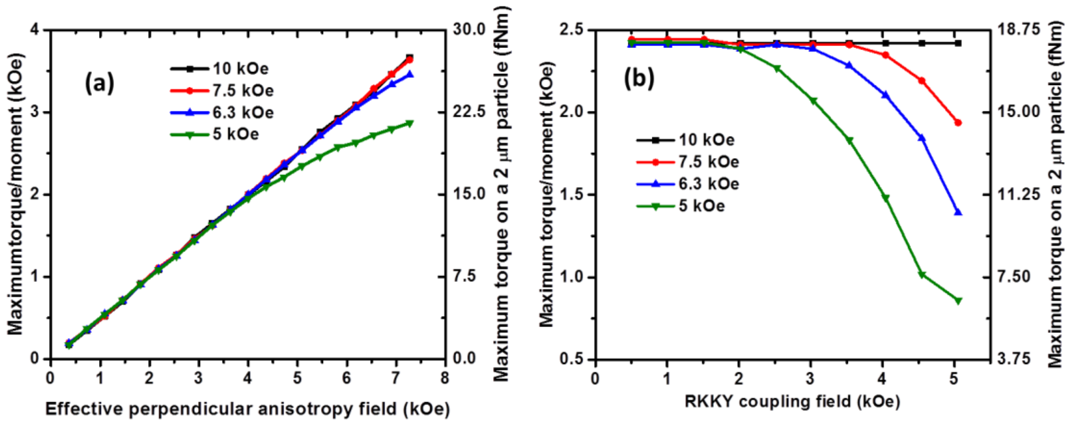

Figure S6. (a) Torque as a function of effective perpendicular anisotropy assuming J = 0.25 erg/cm2 and other parameters as extracted from figure S4 (a). (b) Torque per unit moment as a function of RKKY coupling field and other parameters extracted from figure S4 (a).

6. Retarding torques in a fluid

We assume a quasi-static Stoner-Wohlfarth like model to discuss the torques exerted by the two types of particles in this work. This is only applicable if the retarding effect of the fluid drag is negligible on the scale of the magnetic torques that the particle experiences. The maximum torque in the case for the Py and CoFeB/Pt discs in this study is of the order of 10^{-14} Nm. The microdiscs will be in the viscous flow regime of the cytosol inside the cell, which we take to have a viscosity of 0.04 Pa.s [S3]. The Stokes drag on a thin disc in this regime is then $F_d = \frac{32}{3}\eta a^2 \omega$ and the retarding torque is $T_d = \frac{32}{3}\eta a^3 \omega$ [S4]. Using $\omega = 40\pi$ (20 Hz) we can estimate the retarding torque for our particles in cytosol at 5×10^{-17} Nm. The large peak torques on the magnetic particles in the 10 kOe applied field are over two orders of

magnitude greater than the relevant torque arising from fluid drag, and thus a Stoner-Wohlfarth like quasi-static approach is adequate to model the system.

7. Hyperthermia

Magnetic hyperthermia is a well-established technique for killing cancer cells and works by small magnetic particles heating under a rapidly oscillating magnetic field. It is useful, therefore, to confirm that heating processes are unlikely to be a major cause of cell death in these experiments. We do not expect there to be notable hysteretic losses in these samples, under a rotating field the largest energy loss will be due to frictional losses whilst rotating in the fluid. For a disc rotating about a high symmetry axis the drag torque is given by $T_{drag} = \frac{32}{3}\eta a^3 \omega$, where η is the dynamic viscosity, a the radius of the disc and ω the angular velocity [S4]. The power dissipated is given by $P = T_{drag}\omega$, which is equal to 6.7×10^{-15} W/particle. Magnetic hyperthermia is usually quoted as a specific absorption rate in W/g which for the CoFeB/Pt particles gives a value of around 10 mW/g of magnetic material. This is very small compared to the usual range of 100-1000 W/g used in magnetic hyperthermia experiments [S5,S6], and is therefore unlikely to be an important source of cell death.

8. Dipole fields

Chaining

When magnetized, the particles will produce dipole fields which can cause them to agglomerate. This is true of all magnetic particles, however, it is important for the applications investigated here that they disagglomerate when the magnetic field is removed. This is a problem for the Py vortex particles which have relatively high susceptibilities at low fields and tend to remain agglomerated. In order to prevent this, the CoFeB/Pt particles use RKKY coupling. Below a certain applied field, either external or from the dipole field from other particles, the coupled layers will switch to align antiparallel to each other. This will lead to no net moment and the effect of this coupling is also to give a low susceptibility at low field. Note in figure S4(a), for instance, that the susceptibility of the CoFeB/Pt particles at low fields is greater along the hard axis than the easy axis. Since the particles should disagglomerate this gives a lower bound to the RKKY coupling strength that can be used. In order for the particles to unchain quickly a high RKKY coupling field is needed so that particles in the middle of a chain of particles go to their zero remanent state when the applied magnetic field goes to zero. The dipole field on one particle in the centre of a chain is plotted as a function of chain length in figure S7 and shows that RKKY coupling strengths of over 1.5 kOe would be required in order to get long chains to rapidly disaglommerate. In our particles the coupling field used is around 2 kOe which means these particles should indeed not remain agglomerated after the external field is removed.

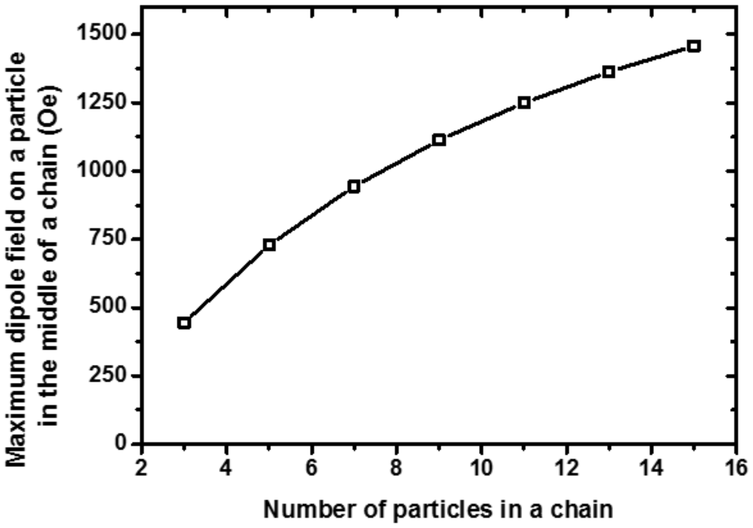

Figure S7. Maximum dipole field on a particle in the middle of a chain as a function of chain length.

Initial forces on particles after magnetization

There is a further interesting estimate that can be made from the dipole fields. When the field is applied and the particles magnetized they will experience a force that will cause them to agglomerate. This force is roughly given by the gradient of the applied field and the moment. Assuming the gradient comes from the dipole field of a neighbouring magnetized particle the force is as high as a few nanoNewtons just before the particles touch. This is a large force and will be of the same order of magnitude for both the Py vortex and CoFeB/Pt particles. This would damage the cells [S7]. The fact that the CoFeB/Pt particles show much greater cell killing than the Py vortex particles suggests that dipole induced forces are not the main mechanism for inducing cell death in the CoFeB/Pt, although this cannot be ruled out in the case of the Py vortex particles.

Next page:

Figure S8. Larger optical images parts of which are used to create the figure shown in the main text.

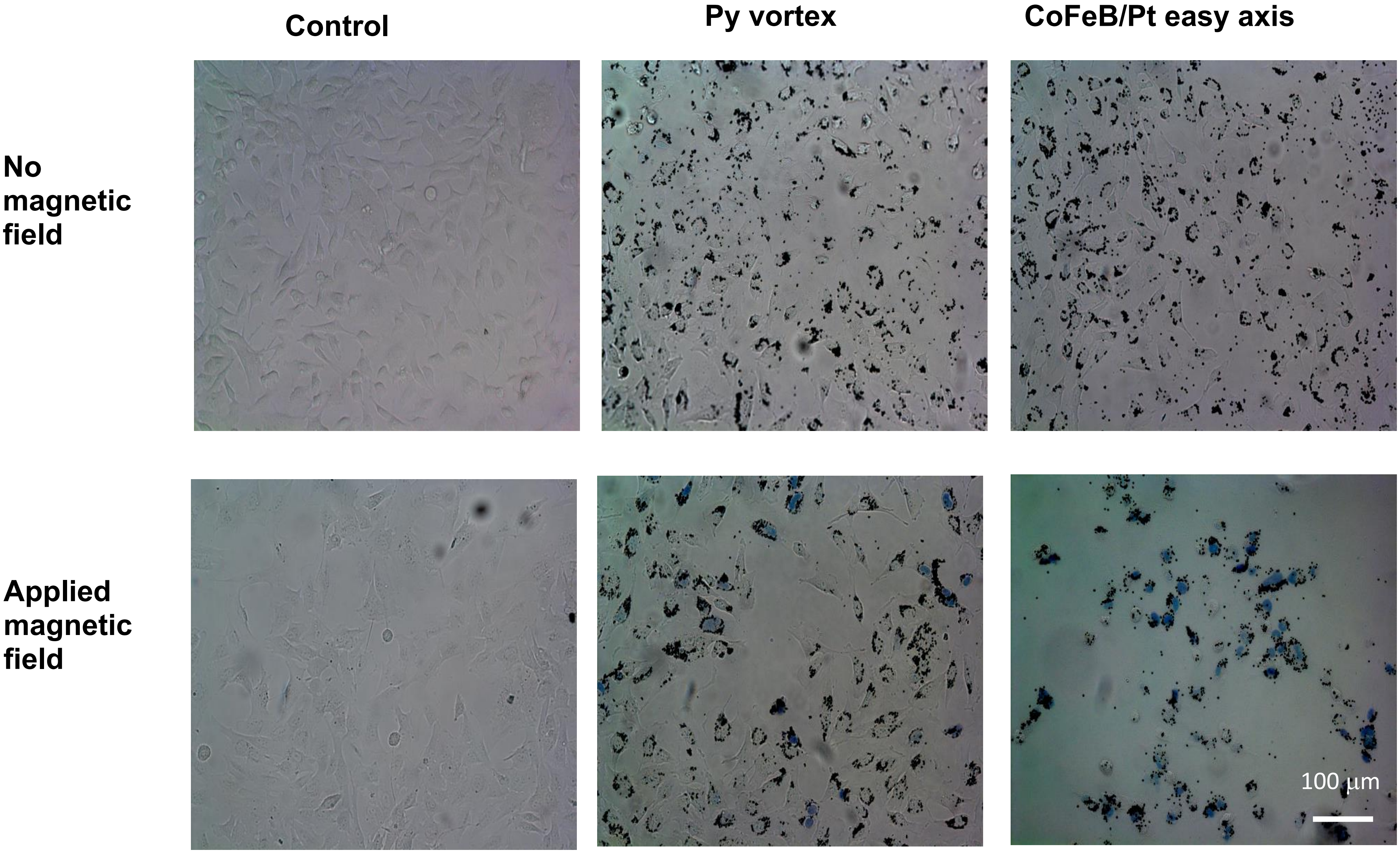
Control
Py vortex
CoFeB/Pt easy axis
No magnetic field
Applied magnetic field
100 μm

References

[S1] Rotating magnetic field induced oscillation of magnetic particles for *in vivo* mechanical destruction of malignant glioma, Cheng, Y., Muroski, M.E., Petit, D.C.M.C., Mansell, R., Vemulkar, T., Morshed, R.A., Han, Y., Balyasnikova, I.V., Horbinski, C.M., Huang, X., Zhang, L., Cowburn, R.P., Lesniak, M.S., Journal of Controlled Release, 223, 75-84 (2016)

[S2] In-plane and out-of-plane anisotropic magnetoresistance in Ni80Fe20 thin films, Th. G. S. M. Rijks, S. K. J. Lenczowski, R. Coehoorn and W. J. M. de Jonge, Phys. Rev. B 56, 362 (1997)

[S3] Comparative Analysis of Viscosity of Complex Liquids and Cytoplasm of Mammalian Cells at the Nanoscale, Tomasz Kalwarczyk, Natalia Ziębacz, Anna Bielejewska, Ewa Zaboklicka, Kaloian Koynov, Jędrzej Szymański, Agnieszka Wilk, Adam Patkowski, Jacek Gapiński, Hans-Jürgen Butt, and Robert Hołyst, Nano Letters 11, 2157-2163 (2011)

[S4] The Stokes resistance of an arbitrary particle, H Brenner, *Chemical Engineering Science*, **18**, 1 (1963)

[S5] Magnetic nanomaterials for hyperthermia-based therapy and controlled drug delivery, C. S. S. R Kumar and F. Mohammed, Adv Drug Deliv Rev., 63, 789–808 (2011)

[S6] Optimal Size of Nanoparticles for Magnetic Hyperthermia: A Combined Theoretical and Experimental Study, B. Mehdaoui, A. Meffre, J. Carrey, S. Lachaize, L.-M. Lacroix, M. Gougeon, B. Chaudret and M. Respaud, Adv. Funct. Mater. 21, 4573–4581 (2011)

[S7] Biofunctionalized magnetic-vortex microdiscs for targeted cancer-cell destruction, D.H. Kim, E.A. Rozhkova, I.V. Ulasov, S.D. Bader, T. Rajh, M.S. Lesniak, V. Novosad, Nature Materials, 9, 165–171 (2010)
